# Correlation between Burning Mouth Syndrome and Anxiety in the Elderly Inmates of Sanitaria in Tehran

**DOI:** 10.5681/joddd.2010.011

**Published:** 2010-06-24

**Authors:** Sedighe Bakhtiari, Hamid Reza Khalighi, Somayyeh Azimi, Kaveh Alavi, Hasan Ayoobi Valoogerdi, Zahra Namazi

**Affiliations:** ^1^ Assistant Professor, Department of Oral Medicine, Shaheed Beheshti University of Medical Sciences, Tehran, Iran; ^2^ Assistant Professor, Department of Oral Medicine, Qazvin University of Medical Sciences, Qazvin, Iran; ^3^ Post-graduate Student, Department of Psychology, Iran University of Medical Sciences, Tehran, Iran; ^4^ Dentistzz Private Practice

**Keywords:** Burning mouth syndrome, anxiety, state, trait

## Abstract

**Background and aims:**

Burning mouth syndrome (BMS) is a chronic pain disorder characterized by a chief complaint of oral burning/pain with no clinically observable oral mucosal lesions. The prevalence of BMS has been reported to be 2.5-5.1% in the general population and several psychological disorders have been reported as associated or predisposing factors for BMS. The aim of this study was to determine the correlation between BMS and anxiety in the elderly residents of sani-taria in Tehran, Iran.

**Materials and methods:**

In a cross-sectional analytical study, 50 patients with BMS were included along with 50 healthy individuals as controls. Inclusion criteria were: age over 60 years, burning sensation in the mouth, normal oral mu-cosa, absence of diabetes, satisfactory prosthesis, absence of iron deficiency and other nutrients, and no heavy smoking habits. Similar inclusion criteria were considered for the control group without any oral complaints. Burning/pain severity was assessed by a 100-mm visual analog scale and the severity of anxiety (state, trait, and total) was determined by Cattell's Anxiety Scale. Data was analyzed by t-test and Pearson's correlation coefficient.

**Results:**

Individuals with BMS assessed their burning/pain severity to be 27.4 mm (95% CI=25.2 - 29.6 mm). Standard-ized total anxiety scale in individuals with and without BMS (±SE for mean) was 5.9±0.2 and 4.6±0.3 with significant dif-ferences (p=0.001). Similar significant differences were observed in state and trait anxiety between the two groups.

**Conclusion:**

It seems that both state and trait anxiety are associated with burning mouth syndrome.

## Introduction

Burning mouth syndrome (BMS) is a chronic pain disorder characterized by a chief complaint of oral burning/pain with no clinically observable lesions in the involved oral mucosa.^1 - 4^ Different terms are used for its identification, including stomatopyrosis (mouth burning), glossopyrosis (tongue burning), stomatodynia (mouth pain), glossodynia (tongue pain) and oral dysesthesia.^4^ The syndrome usually affects 37-78 year-old people with a mean age of 60;^1 , 3 , 5 , 6^ the female-to-male ratio is reported to be 7:1. It mainly involves the tongue with an overall prevalence of 2.5%-5.1%.^4^Psychological factors are believed to have an important role in BMS etiology.^2 - 4^ Psychological factors account for BMS symptoms in more than 50% of patients.^2 , 7^ In addition, most BMS patients demonstrate higher grades of anxiety and depression.^4 , 8 , 9^ Some investigations have reported that depression is the most prevalent psychological disorder in these patients with a prevalence of 31%.^10 , 11^ However, most studies have concluded that anxiety is the main psychological etiologic factor, which is the most difficult obstacle to treatment.^7 , 11 , 12^ Management of secondary BMS must include the elimination of predisposing factors such as stimulants in dietary habits (tobacco, alcohol, etc), nutritional deficiencies, and denture mechanical injuries etc. However, in primary BMS, management aims at decreasing pain and burning sensation as a supportive modality using local capsaicin, mouthwash and chlordiazepoxide and amitriptyline when it is associated with anxiety and depression.^4 , 8^ In addition to burning sensation, burning mouth syndrome alters patient's daily and social activities, resulting in sleep disorders, behavioral changes, impatience, personality and psychological disorders and even cancer phobia as reported in 20% of cases;^13^ therefore, reassuring the patients that there is no relation between cancer and BMS is of utmost importance. Little et al^14^ evaluated anxiety in BMS patients; the study showed that these patients have greater anxiety in the range of the upper 25% of the population. The accurate diagnosis of the etiological factor is helpful for treatment.^4^ In most cases, psychological evaluations are ignored, leading to inaccurate diagnosis and management.^4^ Sardella et al^15^ compared the initiating or precipitating factors in BMS patients and their matched controls. No significant differences were observed between the two groups except for anxiety and depression; therefore, the authors suggested that these two factors might be the main etiology involved in BMS. Lamey et al^1^ studied vulnerability and presenting symptoms in burning mouth syndrome and showed higher HADS (Hospital Anxiety and Depression Scale) in BMS patients compared to the controls. Al-Quran^2^ compared 5 personality traits (conscientiousness, agreeableness, openness, extraversion, neuroticism) according to NEOPI-R questionnaire in the BMS and control groups and showed higher grades in these aspects except for agreeableness in the BMS group. Carlson et al^15^ showed no significant differences between the general population and BMS patients in depression, anxiety and somatization according to McGill (pain questionnaire) MPI and Revised Symptom Checklist (SCL-90R) scale. The present study was performed to determine the relationship between BMS and anxiety disorder in elderly residents of sanitaria in Tehran, Iran in 2008.

## Materials and methods

In a cross-sectional analytical study, 50 patients with burning mouth syndrome (BMS) were selected by stratified sampling technique from 28 sanitaria in Tehran. The sample size was determined by a pilot study on 10 patients, from which standard deviations of 8 cases were calculated for anxiety score; this sample size showed a score difference of 4.5 between the two groups at α=0.05 and β=0.2. Fifty healthy individuals were selected as the control group. Equal numbers of patients and controls were studied in each sanitarium. Burning sensation or pain in the mouth with no observable oral mucosal lesion was considered burning mouth syndrome (BMS). The case group members were questioned about the burning characteristics, including location, duration and previous management, and examined for any oral lesion for exact diagnosis of BMS. In both groups, individuals over 60 were included. Denture-wearing was not a criterion for excluding patients from the study, but only patients who were satisfied with their dentures and had no lesions in the denture-bearing areas were included. Patients with a medical history of cardiovascular diseases (e.g. hypertension and heart failure), diabetes mellitus, anemia and nutritional deficiencies and previous cerebrovascular disease and individuals with dehydration (determined by mucosal dryness) were excluded and substitution was carried out appropriately. Other systematic diseases were not considered exclusion criteria. Smoking history was taken. Heavy smokers (more than 20 pack/year) were excluded. Drug history was taken, based on individual record file.

To evaluate state and trait anxiety Cattell Anxiety Scale was completed by patients without any time limitations. This questionnaire includes 40 questions (20 items for state anxiety and 20 items for trait anxiety). The questions were asked with mild similar tone of illiterate participants and their answers were recorded. Raw scores and standardized scores for the Iranian population were calculated.

The severity of mouth burning or pain was determined by a 100-mm VAS (visual analogue scale),^16^ in which 0 demonstrates no pain and the 100 shows severe intolerable pain.

Statistical analysis was carried out using SPSS10 software (Chicago, Il.). The baseline data, including sex, age, denture wearing and medication use were compared between the case and control groups using Student’s t-test and chi-square test, as indicated. Anxiety scores between the groups were compared using Student’s t-test. Correlation between anxiety scores was calculated by Pearson’s correlation coefficient. In all the cases, a p-value of <0.05 was considered as statistically significant.

## Results

The case group consisted of 6 males and 44 females (12% males), while the control cases comprised 11 males and 39 females (22% males) (p=0.183). The mean ages (±SD) of 69.7±10.0 and 70.9±9.8 years were recorded in the case and control groups, respectively (p=0.545). The frequency of complete denture-wearing patients were 41 (82%) and 45 (90%) in the BMS and control groups, respectively (p=0.249). Frequency of individuals on any group of medications evaluated (beta-blockers and other antihypertensive medications, including diuretics, benzodiazepines, non-steroidal anti-inflammatory drugs, anti-parkinsonian medications, digitals, and any medications with anticholinergic properties) was not statistically different between the case and control groups. In the case group, 48% of the subjects had burning or pain sensation less than half a day, while no patients had all-day pain. Tongue and gingiva were burning sensation locations in most patients with 44% and 34% frequencies, respectively. The mean pain scale, based on a 100-mm VAS, was 27.4 mm (SD=7.8, 95% CI=25.2–29.6 mm), ranging from 10 to 50 mm.

As shown in Table 1, state and trait anxiety scores were higher in the case group. The correlation of total anxiety scores is also shown in Table 2; the results indicated significant correlation of raw and normalized trait and state anxiety scores with each other, but not with patients' age or severity of pain in BMS individuals.

**Table 1:** Scores of state, trait and total anxiety in the case and control groups according to Cattel Anxiety Scale

anxiety			case			control		P value
		mean±SE	median	range	mean±SE	median	range	
Raw scores	State	22.2±0.7	20	13-33	18.3±0.9	18.5	6-34	0.001
	Trait	20.2±0.7	20	9-33	17.5±0.7	18	9-26	0.007
	Total	42.3±1.0	42	29-63	35.8±1.4	36.5	17-59	<0.001
Normalized scores	State	6.1±0.3	6	3-10	4.7±0.3	5	0-10	0.001
	Trait	5.4±0.3	5	1-10	4.3±0.3	4.5	0-8	0.005
	Total	5.9±0.2	6	3-10	4.6±0.3	5	1-10	0.001

**Table 2:** Pearson's correlation coefficients between anxiety scores according to Cattell Anxiety Scale and age, and with severity of pain (based on a 100-mm visual analog scale( in the cases

	Raw scores		Normalized scores	
	r	p	r	p
Trait anxiety & State anxiety	0.424	<0.001	0.431	<0.001
State anxiety & age	-0.050	0.622	-0.046	0.648
Trait anxiety & age	-0.123	0.224	-0.116	0.251
Total anxiety & age	-0.098	0.331	-0.100	0.324
State anxiety & severity of pain	-0.053	0.714	-0.031	0.831
Trait anxiety & severity of pain	-0.074	0.610	-0.058	0.688
Total anxiety & severity of pain	-0.086	0.551	-0.064	0.658

## Discussion

Burning mouth syndrome is a medical challenge with no known etiology despite various studies carried out. Karshan et al^16^ reported it is an anxiety disorder.

The results of the present study showed highly significant differences in anxiety scores between the case and control groups (5.9±1.5 vs. 4.6±2.2, p<0.001). Some studies have emphasized the correlation between anxiety and BMS, but it must be noted that most of these studies are cross-sectional and cannot show the time sequence exclusively; therefore, the causative relationship between anxiety and BMS cannot be established clearly. Therefore, patient history is helpful in determining whether this sensation was before the behavioral change or after that. Furthermore, most patients who have participated in these investigations have been those seeking treatments following severe pain sensation or those who have been seriously concerned about their health. This makes cancer phobia a psychological disorder in BMS patients,^1 , 10 , 18 , 19^ although no cases of this was reported by Sardella et al.^15^

In the present study, trait anxiety scores or anxiety were significantly higher in BMS patients than the controls (20.2±4.6 vs. 17.5±5.1, p=0.007), although this finding does not show anxiety as an etiologic factor for BMS. Due to the nature of such studies, this relationship cannot be clearly established.^20^ As both BMS and anxiety disorders are prevalent diseases, the associated factors may be the result of their interaction with other unknown factors. For example, as BMS and correlated psychological disorders (anxiety and depression) are seen in post-menopausal women, Wardrop et al^21^ suggested hormonal factors to justify the relationships. The role of life stressful events in BMS is not clearly known. Essex et al^22^ concluded that mother's stress in early childhood can make their children more vulnerable when confronted with life events. In this regard, Eli et al^12^ reported a positive relationship between psychological alterations (especially anxiety and depression) and BMS.^12^

The cases indicated more significant use of Benzodiazepines (p=0.017), and calcium (vitamin D) (p=0.028) than the controls. The possible psychological reactions must be noted when calcium ingredients are used.

BMS patients in a study carried out by Lamey et al^1^ showed more diverse diseases than the controls, which forced them to seek treatment and use various drugs; however, in this study the patients were evaluated by screening, not based on BMS problems.

Furthermore, Pilling et al^23^ showed that head and neck region is a common site for unusual sensations without any obvious organic problems.

## Conclusion

The present study showed that both state and trait anxiety are associated with burning mouth syndrome.
